# Transformation of mixed plastic waste: beyond a one-size-fits-all solution

**DOI:** 10.1093/nsr/nwaf488

**Published:** 2025-11-07

**Authors:** Mei-Qi Zhang, Shuheng Tian, Bo Peng, Mingyu Chu, Meng Wang, Ding Ma

**Affiliations:** Beijing National Laboratory for Molecular Sciences, New Cornerstone Science Laboratory, College of Chemistry and Molecular Engineering, Peking University, China; Key Laboratory of Catalysis and Energy Materials Chemistry of Ministry of Education and Hubei Key Laboratory of Catalysis and Materials Science, South-Central Minzu University, China; Beijing National Laboratory for Molecular Sciences, New Cornerstone Science Laboratory, College of Chemistry and Molecular Engineering, Peking University, China; Sinopec Research Institute of Petroleum Processing Co., Ltd, China; Beijing National Laboratory for Molecular Sciences, New Cornerstone Science Laboratory, College of Chemistry and Molecular Engineering, Peking University, China; Beijing National Laboratory for Molecular Sciences, New Cornerstone Science Laboratory, College of Chemistry and Molecular Engineering, Peking University, China; Beijing National Laboratory for Molecular Sciences, New Cornerstone Science Laboratory, College of Chemistry and Molecular Engineering, Peking University, China

## Abstract

This article outlines three strategies to transform mixed plastic waste into fuels and new chemicals, offering a multiple path solution for a circular economy.

Currently, our energy and material systems remain deeply intertwined with fossil resources. This dependency supports the production of transport fuels, chemicals and polymers that are foundational to modern life. However, the unsustainable nature of this model is increasingly apparent. With global plastic production exceeding 400 million tons annually and projected to reach 1.1 billion tons by 2050, the plastics industry alone could consume 20% of global oil production and contribute 15% of carbon emissions [1]. Waste plastics comprise complex mixtures of polymers, additives and contaminants, with lifespans ranging from weeks to decades, leading to a recycling efficiency of less than 10%, achieved primarily through mechanical methods (Fig. 1a, Pathway 1). Although the emerging concept of an electrified refinery that utilizes CO_2_, biomass and plastic wastes as feedstocks to produce fuels and chemicals offers a pathway toward a fossil-free future [2], realizing this vision requires the development of effective recycling technologies capable of reintegrating real-world mixed plastic waste streams into chemical supply chains, which is a crucial step toward addressing resource scarcity and environmental pollution.

**Figure 1.**
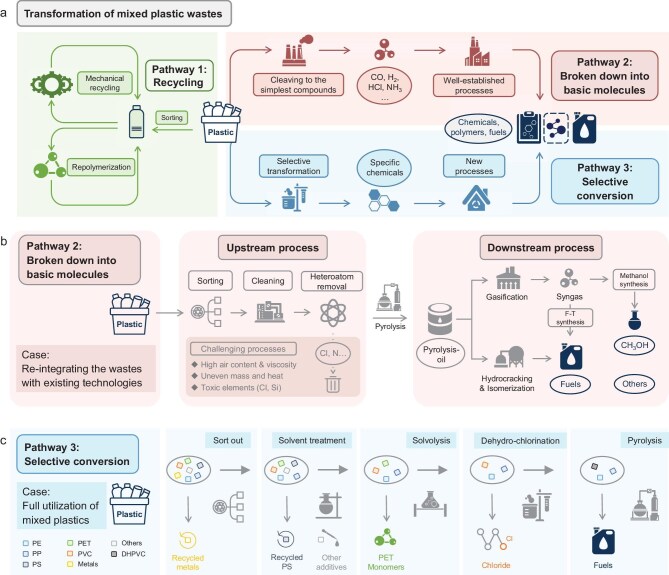
(a) Three possible pathways to re-utilize real-life plastic wastes. (b) Example for Pathway 2: re-integration of plastic waste into well-established industrial processes applying pyrolysis technology. (c). Example for Pathway 3: an anticipated route for making full utilization of plastic packaging waste.

Despite their heterogeneity, plastic mixtures can be conceptually treated as molecular pools—sets of interconnected carbon-, hydrogen-, oxygen-, nitrogen- and halogen-containing compounds. At the same time, they offer valuable chemical reactivity due to the presence of a diverse array of functional groups, including hydroxyl, carboxylic acid, ester and aromatic groups. Recognizing this, we propose two possible strategies for transforming real-life plastic waste: Pathway 2: non-selective deconstruction of plastic mixtures into basic products, such as syngas (CO and H_2_), methane, hydrogen chloride or eventually ammonia, which can serve as feedstocks or fuels for existing industrial infrastructures (Fig. 1b); and Pathway 3: employing selective separation and transformation technologies to convert distinct plastic components into functional monomers, oligomers or high-value chemicals, which can then serve as building blocks for both existing and novel chemical production systems (Fig. 1c).

Retrofitting existing infrastructure to accommodate plastic-derived feedstocks offers a near-term solution, while it imposes stringent requirements on the upstream conversion processes of waste plastics. The upstream process of waste plastics primarily integrates sorting, purification, pyrolysis and heteroatom removal processes, enabling the supply of hydrocarbon feedstocks for existing refinery technology suites such as hydrotreating, catalytic cracking, hydrocracking and isomerization (Fig. 1b). One of the major challenges is the simultaneous removal of heteroatoms (e.g. Cl, O, N) and C–C bond cleavage in complex polymer matrices. Proven strategies for heteroatom removal—including sulfur, nitrogen and oxygen—from organic materials already exist in the petrochemical and biomass industries. Key developed methods, such as hydrotreatment and hydrothermal/solvothermal processing, offer a valuable toolkit for reducing the level of heteroatom contents in plastic waste [3,4]. A viable workaround involves multi-stage or temperature-gradient processes, such as combining low-temperature dehydrochlorination with high-temperature cracking, which has proved to be effective in removing over 99% of chlorine from polyvinyl chloride (PVC)-containing waste streams. However, current methods still struggle to meet refinery-grade purity standards, particularly with respect to residual chlorine and silicon levels [5].

Once upstream processes lower heteroatom contents to below feedstock specifications, the resultant product could be feedstock for further refining, which can therefore enter downstream systems. For example, hydrotreating can refine pyrolysis oils into transport fuels or olefin feedstocks, enabling circularity within the petrochemical value chain. Pilot-scale studies have demonstrated that hydrogenated pyrolysis oils, when blended with petroleum-derived feedstocks, can achieve light olefin yields up to ∼50% in steam crackers [5]. This highlights the potential of a blending strategy to ease the transition toward plastic-derived inputs. Similarly, gasification offers a flexible platform for co-processing mixed plastic waste with other carbonaceous feedstocks (e.g. biomass or coal), producing syngas suitable for downstream applications such as Fischer–Tropsch synthesis or methanol-to-olefins (MTO) routes.

A further frontier lies in element recovery. Many traditional pyrolysis or gasification schemes lose valuable atoms like chlorine or nitrogen, either as waste or low-value byproducts. New strategies seek to co-convert plastic types synergistically. For example, co-processing PVC and polyethylene terephthalate (PET) allows chlorine from PVC to promote PET depolymerization, generating dichloroethane and terephthalic acid as valuable intermediates [6]. Alternatively, complete hydrogenolysis of mixed plastics can yield CH_4_, HCl, H_2_O, and potentially NH_3_ from nitrogen-containing species, offering a chemical route to full-element utilization [7].

Reintegration into existing infrastructure provides a relatively high technology readiness level (TRL) solution, while it often sacrifices the unique structural features of plastics—features that are costly to replicate. To retain and reuse these valuable molecular architectures, a growing body of research is now exploring plastic-specific recovery platforms that apply selective physical, chemical and biological transformations. These strategies typically begin with enhanced sorting, using not just density or conductivity but also spectroscopic tools (e.g. infrared, Raman) and automated imaging systems for high-accuracy material identification [8].

When paired with modular recovery technologies, this enables a more refined approach to waste valorization. For example, plastics can be separated based on their solubility behavior in solvent-based extraction systems. Others may be selectively depolymerized through chemical processes such as solvolysis, oxidation or enzymatic hydrolysis, yielding either monomeric building blocks or reactive intermediates. These processes not only isolate target components but also enable in-line purification or further conversion. Using packaging waste primarily composed of polyethylene (PE), polypropylene (PP) and PET, with minor fractions of PVC, polystyrene (PS), paper and metals, as an example, such waste can be processed through the following stepwise strategy (Fig. 1c): (i) sort and mechanically recycle metals and remove non-plastic debris; (ii) extract PS using solvent-based recycling, simultaneously removing dyes and additives; (iii) depolymerize PET via solvolysis, recovering purified monomers; and (iv) subject remaining polymers to dehydrochlorination and pyrolysis for downstream processing.

This framework aims to maximize the utility of every component, not just recover one dominant polymer at the expense of the rest. Nevertheless, implementing such a system would require either a highly integrated facility or close collaboration among specialized units. While commercial technologies already exist for PS recycling [9], PET solvolysis [10] and polyolefin pyrolysis [11], their systemic integration remains challenging in practical operations, with concerns regarding contamination tolerance and industrial compatibility of different techniques, overall economic benefits and environmental impacts, and so on [12].

Managing real-world plastic waste requires solutions beyond a one-size-fits-all approach. The condition of the waste largely dictates the optimal treatment strategy: post-industrial waste that is relatively clean is suitable for Pathway 1; post-consumer waste with high complexity and level of contamination may be best

handled by Pathway 2 to convert them into feedstocks for existing oil refining systems; specific post-consumer streams like end-of-life agricultural films, textiles or tires have the potential for value increase through Pathway 3. On a more detailed level, the specific micro-structure of the polymers in waste is closely related to the choice of the physical or chemical steps to be a solvent-based process, hydro-conversion or oxidative reaction etc. in a certain pathway. Ongoing exploration of novel reaction systems is yielding more options for producing valuable products using scalable, low-cost, high-performance catalysts.

Policies like the *European Green Deal* and the *China’s 14th Five-Year Plan for Plastic Pollution Control* offer vital institutional support for innovative approaches [13,14]. Beyond setting research and development priorities, they use mechanisms like green finance and carbon pricing to directly influence the economic feasibility of different technological routes. The large-scale application of mixed plastic conversion necessitates a holistic evaluation of technological pathways, integrating policy frameworks with life-cycle environmental impact assessments. From a life-cycle standpoint, Pathway 2 leverages existing refinery infrastructure for rapid deployment but relies on energy-intensive processes like gasification and pyrolysis. In regions with a high share of fossil-based electricity, this can lead to a significantly elevated carbon footprint, making its environmental benefits contingent on the widespread adoption of clean energy. While Pathway 3 aims to preserve polymer value and has the chance to achieve lower carbon emissions;

its full system-level assessment must incorporate the environmental burdens of fine sorting, solvent use, multi-stage purification and so on (Table 1).

**Table 1.** Comparison of pathways for the transformation of mixed plastics.

	Pathway 1	Pathway 2	Pathway 3
Energy consumption	Relatively low for mechanical recycling Moderate to high for chemical recycling	High	Moderate to high
Environmental impact	Generally positive for mechanical recycling Debatable for chemical recycling	Debatable	Debatable
Economic viability	High for clean, single-stream plastics	Low to moderate	Moderate to high
Technological maturity	Low for sorting High for mechanical recycling Low to moderate for chemical recycling	Moderate to high	Low

The pathway toward sustainable plastic valorization must contend with significant variability. Waste stream composition differs across regions and evolves over time due to changes in production practices, consumer behavior, regulations and collection systems. This variability complicates process design, product standardization and life-cycle analysis. As a result, next-generation systems must be built for traceability, adaptability and interoperability across stages—from waste collection to final product.

Achieving this vision will require interdisciplinary collaboration, uniting expertise in analytical chemistry, catalysis, process engineering, data science and policy. Innovations in real-time waste characterization, smart sorting technologies and dynamic process control will also be crucial to closing the loop in plastics use. In this regard, cutting-edge artificial intelligence offers a powerful solution for diagnosing real-life wastes—such as stream composition, polymer structure and additive/contaminant presence—and prescribing optimized recovery processes, achieving a holistic balance between environmental, economic and industrial needs. A full digital twin of a physical plant can be anticipated to run in parallel to enable real-time, self-optimizing operations for maximum yield and minimal energy consumption in the future, which calls for not only the development of advanced artificial intelligence, but also the establishment of reliable datasets containing information about polymer properties, catalytic performance, spectroscopic signatures, lifecycle assessment data and all other relevant aspects.

In conclusion, the sustainable transformation of real-life plastic waste is one of the most complex and urgent challenges of the circular economy. The complementary pathways discussed—integration into existing infrastructure and plastic-specific valorization platforms—represent promising but nascent directions. Much of the required work remains at the laboratory or pilot scale, and key questions regarding economic viability, environmental impact and scalability must still be answered [12]. With focused investment and cross-sector coordination, these strategies can form the foundation of a resilient, low-carbon plastics economy for the future.
